# Supplementary material for: Integrative analysis reveals RNA G-quadruplexes in UTRs are selectively constrained and enriched for functional associations
Source: Nat Commun. 2020 Jan 27;11:527. doi: 10.1038/s41467-020-14404-y (PMC6985247; doi:10.1038/s41467-020-14404-y)
Supplement: Supplementary file 1 — Supplementary Information [file 41467_2020_14404_MOESM1_ESM.pdf]

Integrative analysis reveals RNA G-Quadruplexes in UTRs are selectively constrained and enriched for functional associations

Lee et. al.

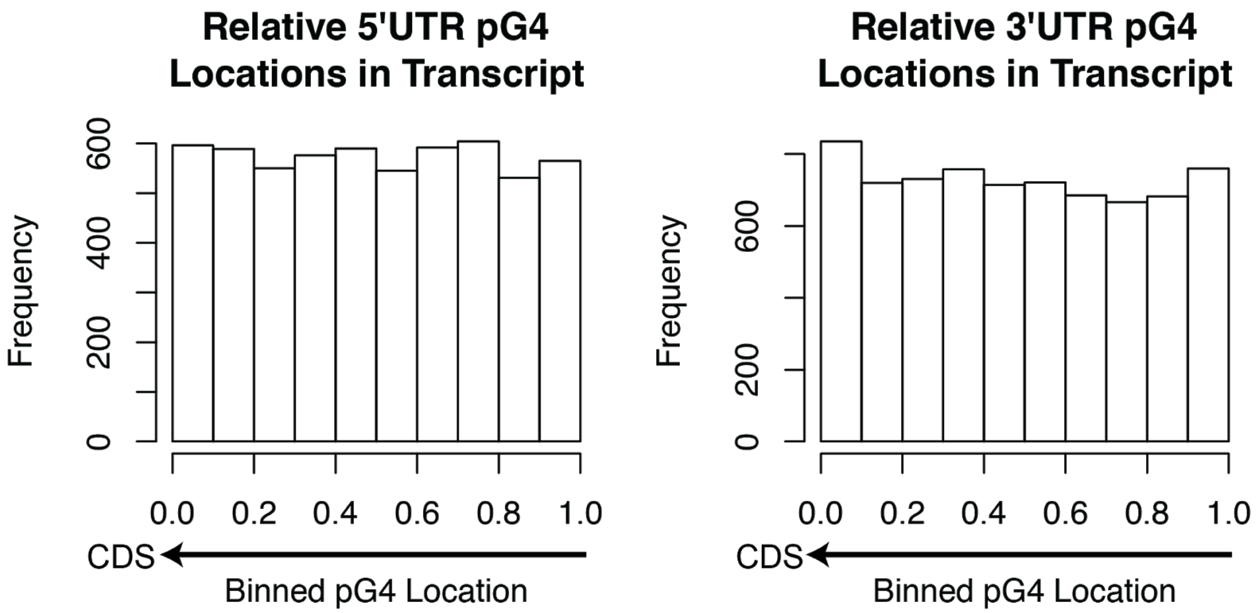

Supplementary Figure 1: Distribution of binned distances for mapped canonical 5’ (left) and 3’ (right) UTR pG4 sequences with respect to protein-coding sequences across pG4-UTR containing mRNA transcripts. The relative locations of pG4 sequences within UTRs are plotted (x-axis), with 0 being adjacent to the coding sequence, and 1 representing the full-length of the annotated UTR away from the CDS. Source data are provided as a Source Data file.

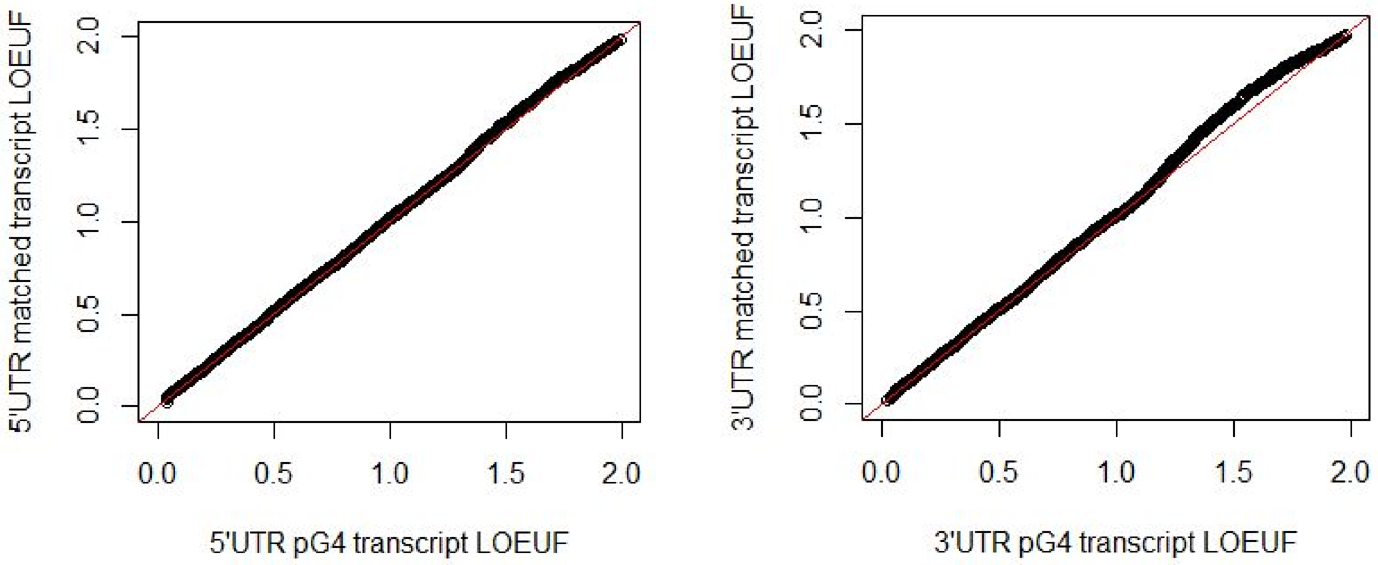

Supplementary Figure 2: Quantile-quantile plot showing matching between pG4 and non-pG4 containing transcripts based on LOEUF scores for 5’UTR (left) and 3’UTR (right) transcripts. Allele frequencies (Figure 1b) and substitutions (Figure 1c) were compared across constraint-matched transcripts using gnomAD’s LOEUF metric to control for the possibility that nearby constrained coding sequences might affect local allele frequency estimates. LOEUF scores for non-G4 transcripts plotted on the X-axis and LOUEF scores for G4-containing transcripts are plotted on the Y-axis.

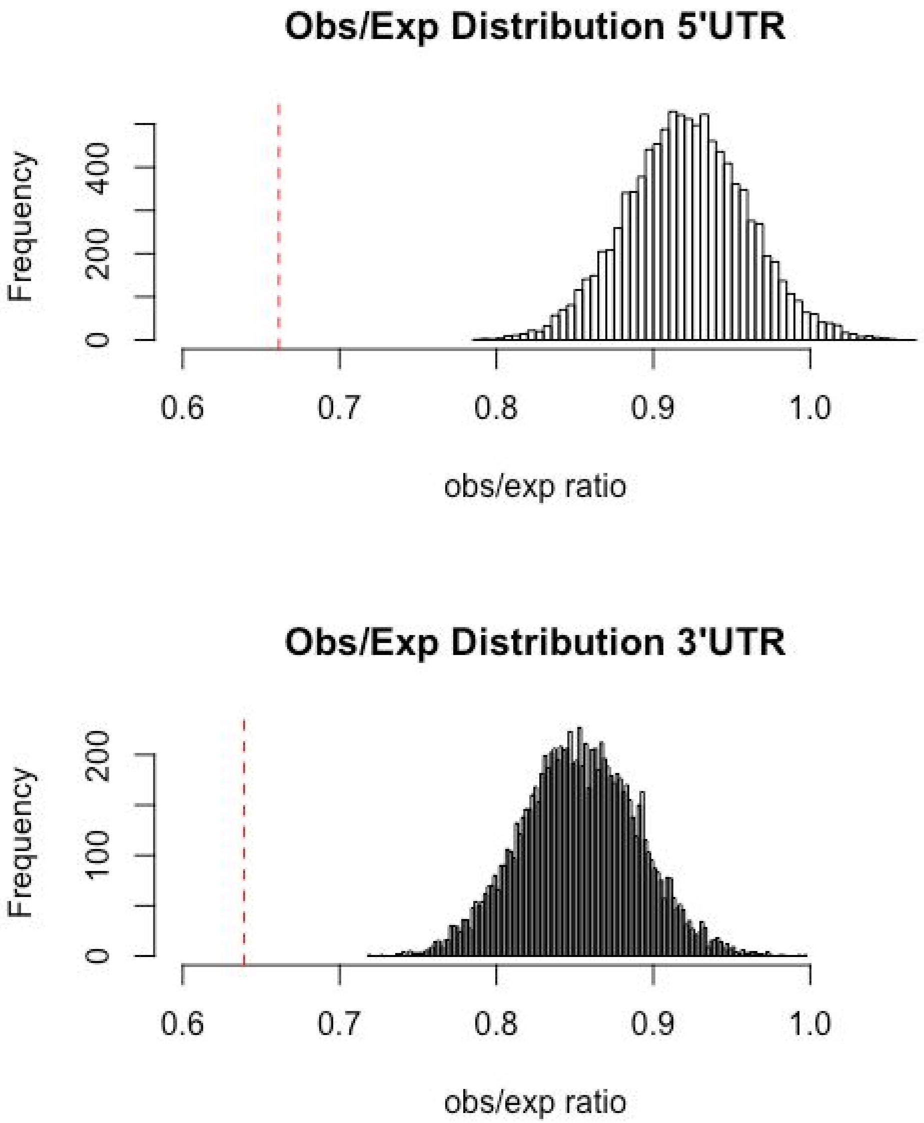

Supplementary Figure 3: The empirical distribution of observed vs. expected number of substitutions across 10,000 bootstrapped 5’ and 3’ UTR regions in the European subpopulation of the 1000 Genomes Project Phase 1 release. Red dotted line indicates the observed vs. expected ratios estimated by applying the noncoding heptamer mutation model across 5’UTR and 3’UTR pG4 sequences respectively. Source data are provided as a Source Data file.

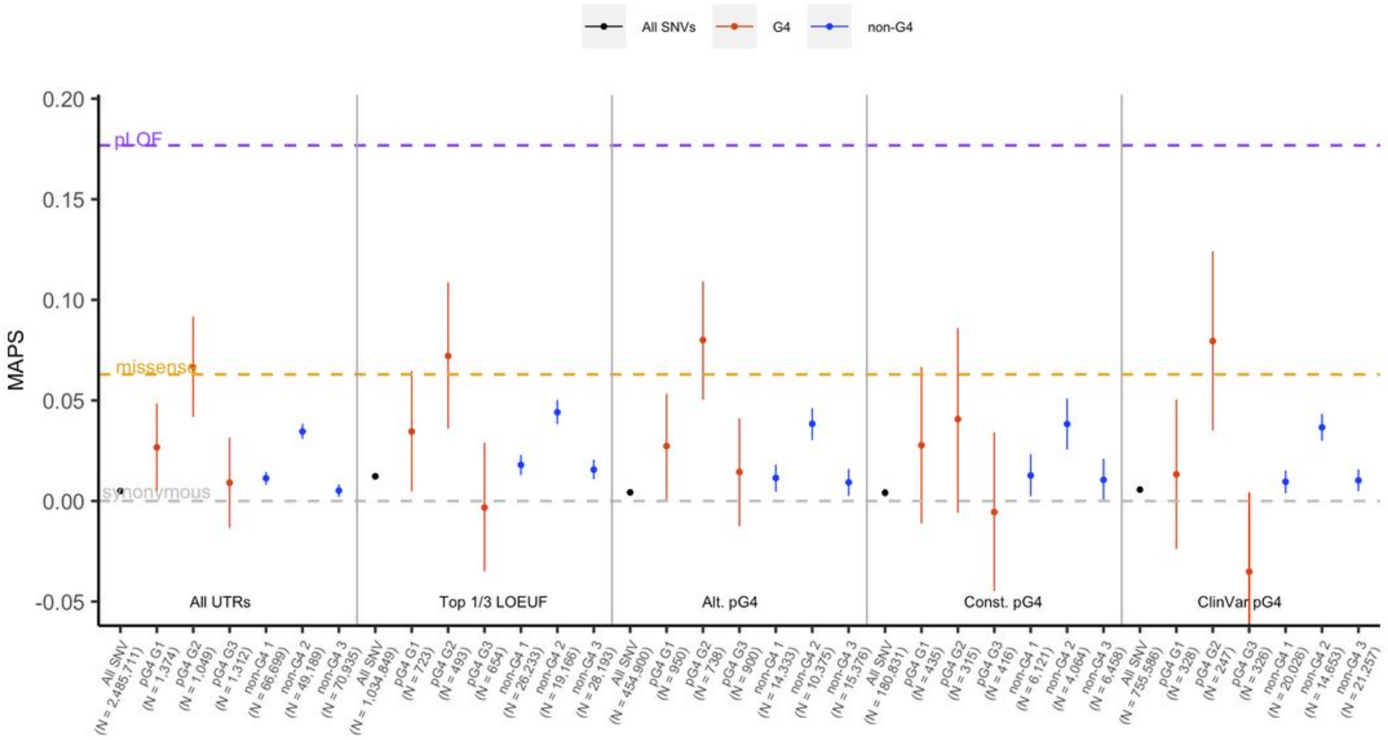

Supplementary Figure 4: MAPS scores for All (black), G4 (red), and non-G4 GGG/CCC (blue) variants across multiple gene sets. Error bars represent 90% CI from 10,000 bootstraps.Top ⅓ LOEUF represent MAPS scores for pG4 versus non-pG4 variants within the top-1/3rd most constrained genes as estimated by the gnomAD LOEUF metric. Alt. pG4 represent alternatively included pG4 sequences while Const. pG4 represent constitutively included pG4 sequences. ClinVar pG4 sequences are those pG4 within UTRs of disease-associated genes in ClinVar. Permutation P-values for the second guanine of each trinucleotide G-tract context compared to non-pG4 G-tracts in UTRs are: P=0.0195 for all UTRs, P=0.1063 for Top ⅓ LOEUF, P=0.0124 for Alt. pG4, P=0.4653 for Const. pG4, and P=0.0579 for ClinVar pG4. Genome-wide MAPS scores for synonymous (grey), missense (orange), and putative loss of function (red) protein-coding variation shown as dotted lines. Source data are provided as a Source Data file.

a

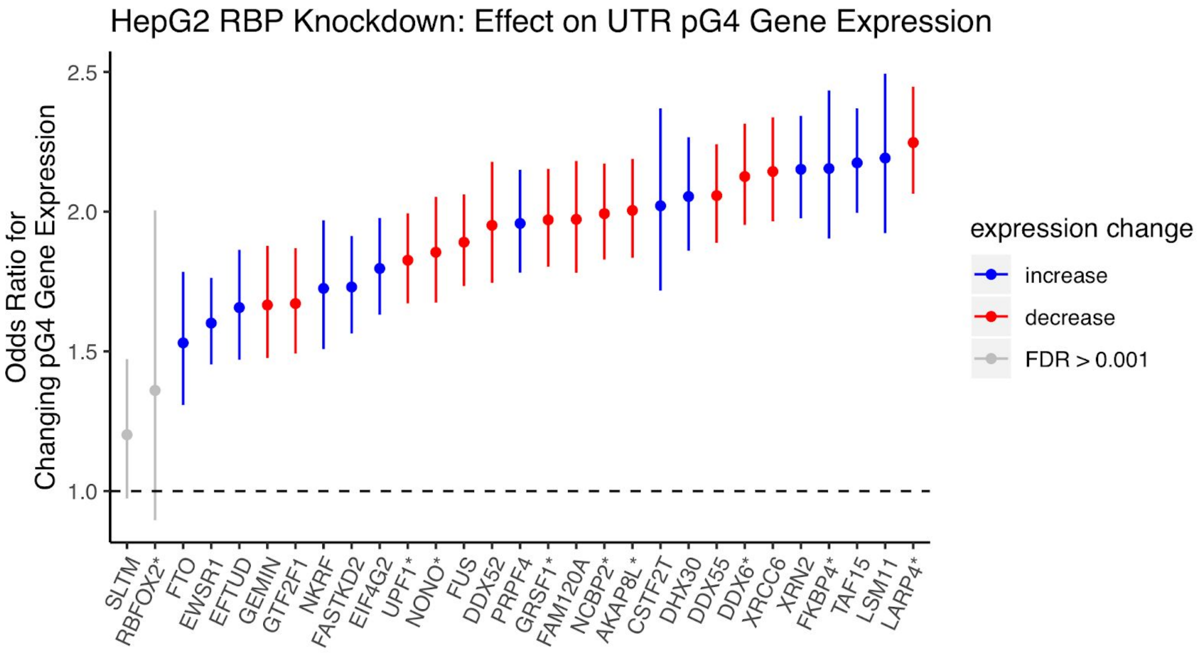

b

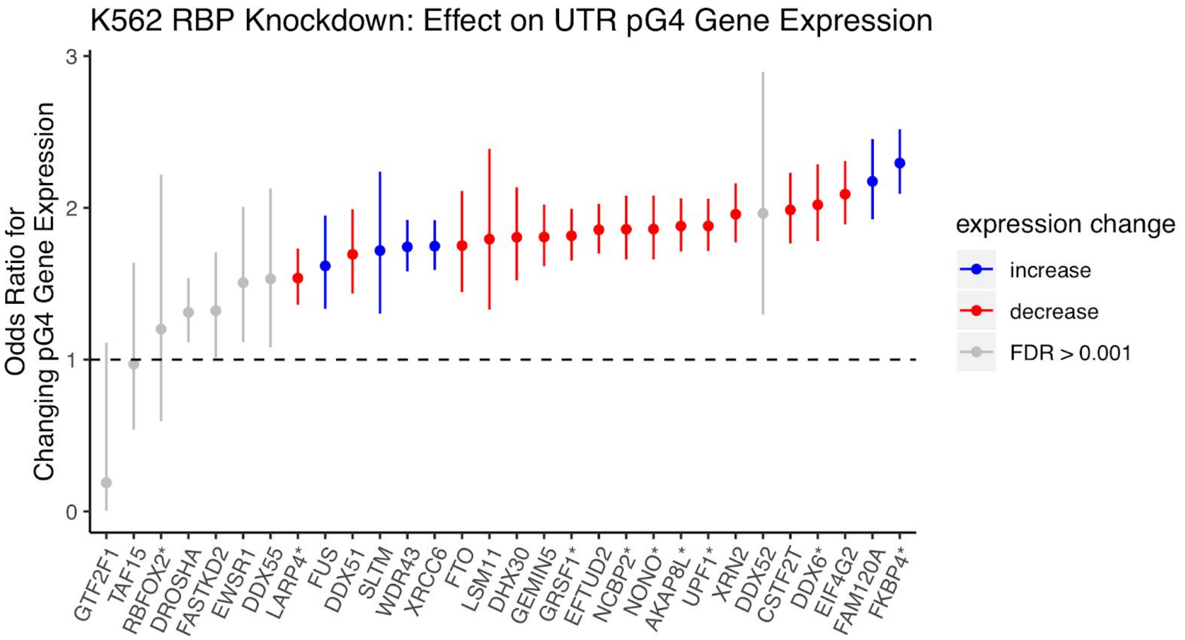

Supplementary Figure 5: Distribution of odds ratio with error bars representing 95% confidence interval for changing gene expression of a pG4 containing gene versus a non-pG4 containing gene with sh-RBP knockdown in ENCODE as determined by Fisher's Exact Test. Results for HepG2 (**a**) and K562 (**b**) are shown. Colors represent a tendency for an RBP-knockdown to increase the expression of pG4 containing genes (blue), decrease their expression (red) or have no effect on changing the expression of pG4 genes (grey) at an FDR < 0.001. Proteins having the same direction on

changing pG4 gene expression across both HpeG2 and K562 cell lines are marked by an asterisk. Source data are provided as a Source Data file.

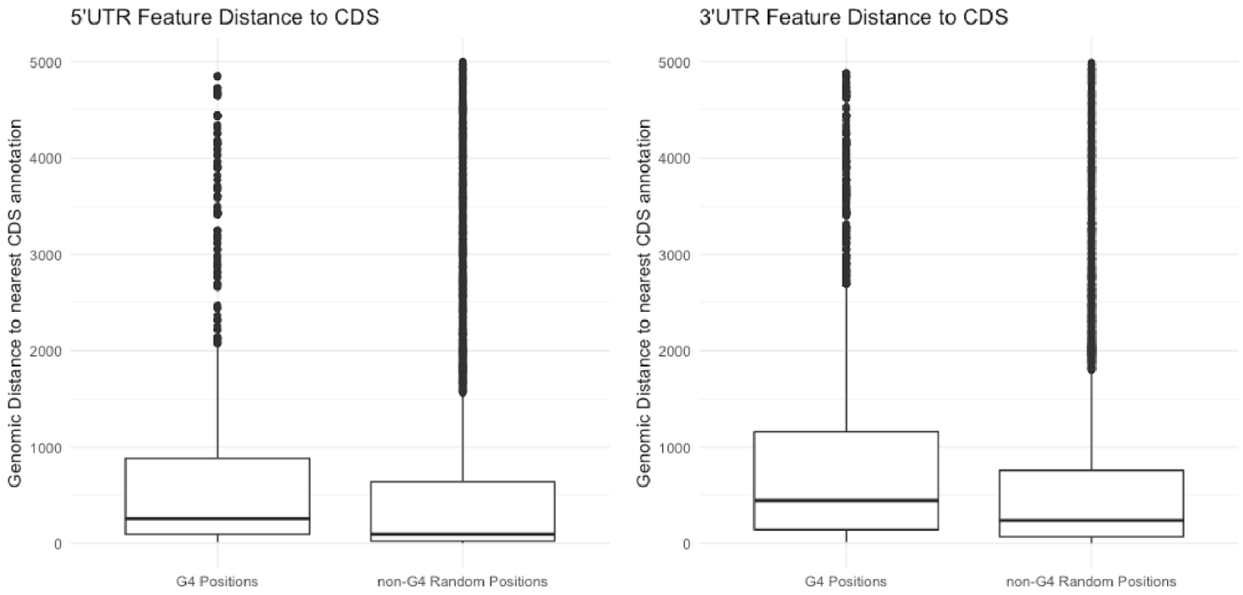

Supplementary Figure 6: Boxplot of distribution of distances between 5' UTR (a) and 3'UTR (b) pG4 sequences and nearest annotated protein-coding exons in ClinVar disease-associated genes showing the 1.5 times the interquartile range and median values. Compared to randomly selected positions within non-pG4 5' and 3' UTRs of ClinVar disease-associated genes, UTR pG4 sequences tend to be located further away from protein-coding exons.

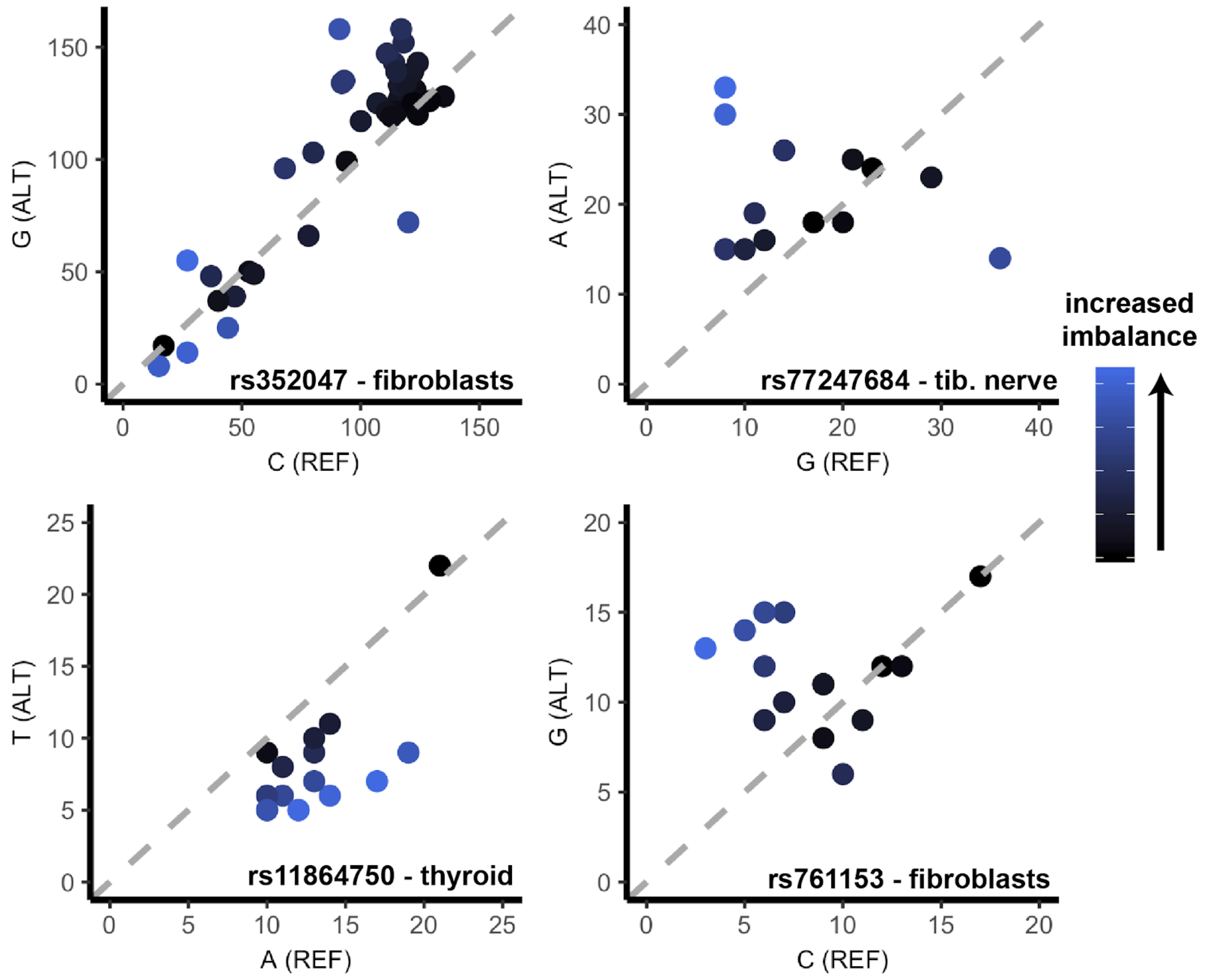

Supplementary Figure 7: Additional common SNPs in high LD ($r^2 > 0.85$ in the 1000 Genomes GBR population) with GWAS tag SNPs exhibiting evidence of allelic imbalance in UTR pG4 sequences. rs352047, rs77247684, and rs761153 affect 3'UTR pG4 sequences. rs11864750 affects a 5'UTR pG4 sequence. Refer to Supplementary Data 5 and 6 for further details on phenotypic associations, study references, and linked tag-SNPs. Source data are provided as a Source Data file.

Supplementary Table 1: cis-eQTL enrichment statistics

Test Set	pG4 N qTLs	pG4 N SNPs	Total	non-pG4 N qTLs	non-pG4 N SNPs	Total	OR	95% CI	P-value (Fisher's Exact Test)
5'UTR Lead cis-eQTLs	34	146	180	1980	17716	19696	2.08	1.39 - 3.05	0.0004132
3'UTR Lead cis-eQTLs	12	146	158	2052	54331	56383	4.96	2.43 - 9.30	1.96E-05
5'UTR Causal cis-eQTLs	22	13	35	1403	1877	3280	2.26	1.09 - 4.91	0.02429
3'UTR Causal cis-eQTLs	10	10	20	1632	6963	8595	4.27	1.59 - 11.44	0.001752
5'UTR Nominal cis-eQTLs	129	413	542	9588	45955	55543	1.5	1.22 - 1.83	0.000126
3'UTR Nominal cis-eQTLs	103	259	362	22032	163582	185614	2.96	2.33 - 3.73	< 2.2e-16
5'UTR RBP cis-eQTLs	30	84	114	1478	7028	8506	0.91	0.58 - 1.38	0.7615
3'UTR RBP cis-eQTLs	103	259	362	2607	17720	20327	2.43	1.54 - 3.73	0.0001083
5'UTR Nominal cis-eQTLs (shared pG4 removed)	120	413	542	9588	45955	55543	1.39	1.13 - 1.71	0.001878
3'UTR Nominal cis-eQTLs (shared pG4 removed)	100	259	362	22032	163582	185614	2.87	2.25- 3.63	2.48E-16

Supplementary Table 2: ENCODE RBP binding enrichment statistics

HepG2 5'UTR pG4-enriched RBPs					
rbp	p.val	bh.p.val	g4_peaks	all_peaks	peak_ratio
PRPF4	3.27E-93	9.89E-93	307	1224	0.25081699
GTF2F1	5.85E-69	1.73E-68	181	594	0.3047138
FAM120A	8.68E-41	2.52E-40	130	506	0.256917
RBFOX2	1.74E-22	4.96E-22	75	311	0.24115756
CSTF2T	2.51E-22	7.02E-22	169	1122	0.15062389
GRSF1	1.94E-18	5.31E-18	44	143	0.30769231
XRN2	2.38E-15	6.41E-15	50	211	0.23696682
FASTKD2	2.80E-10	7.40E-10	26	97	0.26804124
NKRF	1.64E-07	4.27E-07	49	330	0.14848485
FKBP4	2.23E-07	5.70E-07	24	114	0.21052632
FTO	2.84E-07	7.13E-07	31	172	0.18023256
EFTUD2	5.96E-07	1.47E-06	85	719	0.11821975
NCBP2	1.09E-05	2.65E-05	212	2312	0.0916955
SLTM	4.66E-05	0.00011131	17	91	0.18681319
PCBP2	9.08E-05	0.00021324	53	456	0.11622807
DGCR8	0.0002477	0.00057281	19	121	0.15702479
UPF1	0.00052629	0.00119832	21	147	0.14285714
DDX6	0.0064016	0.01435509	4	18	0.22222222
CDC40	0.01232967	0.02723569	9	64	0.140625
TAF15	0.01318996	0.02870755	3	14	0.21428571
K562 5'UTR pG4-enriched RPBs					
rbp	p.val	bh.p.val	g4_peaks	all_peaks	peak_ratio
GTF2F1	9.60E-69	4.58E-68	144	513	0.28070175
CSTF2T	6.32E-35	2.92E-34	130	783	0.1660281
FASTKD2	1.43E-16	6.42E-16	26	76	0.34210526

NCBP2	3.32E-16	1.45E-15	47	246	0.19105691
RBFOX2	3.10E-13	1.31E-12	31	140	0.22142857
GEMIN5	3.93E-12	1.62E-11	113	1164	0.09707904
XRN2	6.11E-12	2.44E-11	37	212	0.1745283
DROSHA	5.43E-11	2.12E-10	75	683	0.10980966
FAM120A	1.35E-09	5.12E-09	25	131	0.19083969
AKAP8L	2.44E-09	9.02E-09	26	144	0.18055556
FUS	6.18E-08	2.23E-07	30	210	0.14285714
EWSR1	4.47E-07	1.57E-06	42	378	0.11111111
FTO	7.20E-07	2.48E-06	35	295	0.11864407
DDX51	2.55E-06	8.58E-06	14	73	0.19178082
XRCC6	1.34E-05	4.40E-05	14	83	0.1686747
DDX52	4.32E-05	0.00013891	13	81	0.16049383
DDX6	9.10E-05	0.00028641	20	167	0.11976048
PHF6	0.00022345	0.00068897	8	43	0.18604651
AATF	0.00190863	0.00576485	8	57	0.14035088
SLTM	0.00400365	0.0118508	14	139	0.10071942
RPS11	0.0071914	0.02046783	1	3	0.33333333
SF3B1	0.0071914	0.02046783	1	3	0.33333333
UPF1	0.01364907	0.03811437	13	145	0.08965517
EFTUD2	0.01404575	0.03849577	29	393	0.07379135
HNRNPU	0.01505849	0.04052103	2	11	0.18181818
HepG2 3'UTR pG4-enriched RPBs					
rbp	p.val	bh.p.val	g4_peaks	all_peaks	peak_ratio
PRPF4	2.55E-119	8.16E-119	165	392	0.42091837
FAM120A	2.16E-75	6.77E-75	179	814	0.21990172
GTF2F1	2.55E-60	7.82E-60	82	194	0.42268041
GRSF1	2.88E-22	8.64E-22	55	275	0.2
RBFOX2	4.87E-16	1.43E-15	53	348	0.15229885

CSTF2T	6.19E-12	1.78E-11	53	437	0.12128146
UPF1	6.34E-09	1.79E-08	394	6973	0.05650366
NKRF	1.77E-07	4.91E-07	20	129	0.15503876
XRN2	2.58E-06	7.00E-06	10	45	0.22222222
FASTKD2	3.37E-06	8.99E-06	7	23	0.30434783
FUS	1.18E-04	3.09E-04	6	27	0.22222222
FKBP4	3.69E-04	9.50E-04	6	32	0.1875
DDX6	7.63E-04	1.93E-03	22	254	0.08661417
TAF15	1.48E-03	3.66E-03	2	6	0.33333333
SLTM	3.63E-03	0.00886669	5	35	0.14285714
EXOSC5	0.00776956	0.01864695	2	10	0.2
RBM5	0.01614163	0.03810483	3	23	0.13043478
BCCIP	0.01722288	0.04000152	1	5	0.2
EIF3H	0.01783001	0.04075431	26	403	0.06451613
K562 3'UTR pG4-enriched RBPs					
rbp	p.val	bh.p.val	g4_peaks	all_peaks	peak_ratio
GTF2F1	2.40E-110	1.12E-109	124	252	0.49206349
DDX6	9.88E-45	4.45E-44	148	1093	0.13540714
FASTKD2	2.11E-35	9.22E-35	31	47	0.65957447
AKAP8L	2.46E-21	1.04E-20	31	104	0.29807692
CSTF2T	1.89E-20	7.77E-20	61	431	0.14153132
DROSHA	5.71E-13	2.28E-12	25	127	0.19685039
FAM120A	5.41E-11	2.11E-10	206	3654	0.05637657
APOBEC3C	9.35E-11	3.54E-10	52	546	0.0952381
DDX55	8.01E-09	2.96E-08	54	655	0.08244275
LSM11	1.95E-08	6.95E-08	25	201	0.12437811
EWSR1	1.98E-08	6.95E-08	20	136	0.14705882
FUS	2.23E-06	7.66E-06	22	209	0.10526316
SLTM	4.81E-06	1.61E-05	15	117	0.12820513

RBFOX2	1.93E-05	6.31E-05	19	190	0.1
FTO	2.77E-05	8.88E-05	6	26	0.23076923
LARP4	6.67E-05	0.00020867	17	175	0.09714286
DDX52	0.00017633	0.00054026	6	34	0.17647059
TAF15	0.00029431	0.00088293	3	10	0.3
DDX42	0.00043989	0.00129267	2	5	0.4
EIF4G2	0.00044884	0.00129267	19	239	0.07949791
WDR43	0.00272647	0.00769827	3	17	0.17647059
XRCC6	0.00297506	0.00823864	8	83	0.09638554
DDX51	0.00331516	0.00900723	2	9	0.22222222
UPF1	0.00657312	0.01752833	388	9651	0.04020309
XRN2	0.00791694	0.020728	4	35	0.11428571
DHX30	0.01002217	0.02577129	4	37	0.10810811
TROVE2	0.01715277	0.04333331	3	28	0.10714286
NONO	0.01820974	0.04521038	13	207	0.06280193
