# Supplementary material for: Integrative analysis reveals RNA G-quadruplexes in UTRs are selectively constrained and enriched for functional associations
Source: Nat Commun. 2020 Jan 27;11:527. doi: 10.1038/s41467-020-14404-y (PMC6985247; doi:10.1038/s41467-020-14404-y)
Supplement: Supplementary file 3 — Reporting Summary [file 41467_2020_14404_MOESM3_ESM.pdf]

nature research

Corresponding author(s): Louis Ghanem, Yoseph Barash

Last updated by author(s): Dec 10, 2019

Reporting Summary

Nature Research wishes to improve the reproducibility of the work that we publish. This form provides structure for consistency and transparency in reporting. For further information on Nature Research policies, see Authors & Referees and the Editorial Policy Checklist.

Statistics

For all statistical analyses, confirm that the following items are present in the figure legend, table legend, main text, or Methods section.

n/a	Confirmed	
☐	☒	The exact sample size (*n*) for each experimental group/condition, given as a discrete number and unit of measurement
☐	☒	A statement on whether measurements were taken from distinct samples or whether the same sample was measured repeatedly
☐	☒	The statistical test(s) used AND whether they are one- or two-sided<br>*Only common tests should be described solely by name; describe more complex techniques in the Methods section.*
☐	☒	A description of all covariates tested
☐	☒	A description of any assumptions or corrections, such as tests of normality and adjustment for multiple comparisons
☐	☒	A full description of the statistical parameters including central tendency (e.g. means) or other basic estimates (e.g. regression coefficient) AND variation (e.g. standard deviation) or associated estimates of uncertainty (e.g. confidence intervals)
☐	☒	For null hypothesis testing, the test statistic (e.g. *F*, *t*, *r*) with confidence intervals, effect sizes, degrees of freedom and *P* value noted<br>*Give P values as exact values whenever suitable.*
☒	☐	For Bayesian analysis, information on the choice of priors and Markov chain Monte Carlo settings
☒	☐	For hierarchical and complex designs, identification of the appropriate level for tests and full reporting of outcomes
☐	☒	Estimates of effect sizes (e.g. Cohen's *d*, Pearson's *r*), indicating how they were calculated

Our web collection on statistics for biologists contains articles on many of the points above.

Software and code

Policy information about availability of computer code

Data collection	See "Data" for detailed description of how data were collected from the public resources used in this study.
Data analysis	STAR 2.7.0c, R 3.6.1 (see methods for associated R packages used in analyses), Python 3.7

For manuscripts utilizing custom algorithms or software that are central to the research but not yet described in published literature, software must be made available to editors/reviewers. We strongly encourage code deposition in a community repository (e.g. GitHub). See the Nature Research guidelines for submitting code & software for further information.

Data

Policy information about availability of data

All manuscripts must include a data availability statement. This statement should provide the following information, where applicable:

- Accession codes, unique identifiers, or web links for publicly available datasets
- A list of figures that have associated raw data
- A description of any restrictions on data availability

Genetic variants from the Genome Aggregation Database (gnomAD) are publicly available through the gnomAD website (https://gnomad.broadinstitute.org/). Genetic variants from the 1000 Genomes Project are available through their website (http://www.internationalgenome.org/). ENCODE data is available through the ENCODE website (https://www.encodeproject.org/). GTEx v7 eQTLs are publicly available through the GTEx Portal (https://gtexportal.org/). GTEx genotypes and RNA-seq reads are available to authorized users through dbGaP (study accession phs000424.v7.p2). ClinVar variant annotations are available from the National Center for Biotechnology Information website (https://www.ncbi.nlm.nih.gov/).

Field-specific reporting

Please select the one below that is the best fit for your research. If you are not sure, read the appropriate sections before making your selection.

☒ Life sciences ☐ Behavioural & social sciences ☐ Ecological, evolutionary & environmental sciences

For a reference copy of the document with all sections, see nature.com/documents/nr-reporting-summary-flat.pdf

Life sciences study design

All studies must disclose on these points even when the disclosure is negative.

Sample size	Sample size was determined based on the availability of existing data (gnomAD, 1000 Genomes, GTEx, ENCODE)
Data exclusions	Genetic variants from gnomAD and the 1000 Genomes Project Phase 1 Release not passing quality filters (see methods) were excluded from the analysis, as well as insertions and deletions. For cis-eQTL analyses, only SNPs and cis-eQTLs mapping to annotated 5' or 3' UTRs were considered. For analysis of ENCODE CLIP-seq data, ENCODE called peaks with an IDR score of less than 1000 were excluded from downstream analyses.
Replication	We assessed evidence for evolutionary constraint in humans over UTR pG4 sequences by using two large public datasets (gnomAD, 1000 KG), and two different metrics.
Randomization	See methods for how randomization was used to assess confidence intervals for constraint measurements. Otherwise randomization of samples is not relevant to this study since the analysis relies largely on publicly available data.
Blinding	Investigators were not blinded to group allocation during data collection or analysis, as this study relies on the analysis of de-identified, publicly available data sources.

Reporting for specific materials, systems and methods

We require information from authors about some types of materials, experimental systems and methods used in many studies. Here, indicate whether each material, system or method listed is relevant to your study. If you are not sure if a list item applies to your research, read the appropriate section before selecting a response.

Materials & experimental systems

n/a	Involved in the study	
☒	☐	Antibodies
☒	☐	Eukaryotic cell lines
☒	☐	Palaeontology
☒	☐	Animals and other organisms
☒	☐	Human research participants
☒	☐	Clinical data

Methods

n/a	Involved in the study	
☒	☐	ChIP-seq
☒	☐	Flow cytometry
☒	☐	MRI-based neuroimaging
