# Supplementary material for: Integrative analysis reveals RNA G-quadruplexes in UTRs are selectively constrained and enriched for functional associations
Source: Nat Commun. 2020 Jan 27;11:527. doi: 10.1038/s41467-020-14404-y (PMC6985247; doi:10.1038/s41467-020-14404-y)
Supplement: Supplementary file 10 — Description of Additional Supplementary Files [file 41467_2020_14404_MOESM10_ESM.pdf]

Title: Supplementary Data 1:
Description: Table of genes with 5’ or 3’ UTR alternative or constitutive pG4.

Title: Supplementary Data 2:
Description: Statistics for gene ontology analysis of pG4-containing genes.

Title: Supplementary Data 3:
Description: Statistics for gene ontology analysis of RBP-pG4 target genes.

Title: Supplementary Data 4:
Description: Table of ClinVar variants falling within UTR pG4 sequences.

Title: Supplementary Data 5:
Description: Table of GWAS lead variants falling within UTR pG4 sequences.

Title: Supplementary Data 6:
Description: Table of common SNPs linked to GWAS lead variants falling within UTR pG4 sequences
